# Acceptance and commitment therapy for depression in chronic kidney patients: a pilot study

**DOI:** 10.3389/fpsyg.2025.1656929

**Published:** 2025-09-05

**Authors:** Sonia Diéguez, Helena García-Llana, David Lobato, M. Teresa Marín, M. Dolores Arenas, Fabiola Dapena, Francisco Montesinos

**Affiliations:** ^1^Centro de Enseñanza Superior Cardenal Cisneros, Madrid, Spain; ^2^Faculty of Biomedical and Health Sciences, Universidad Europea de Madrid, Madrid, Spain; ^3^Fundación Renal Española, Madrid, Spain

**Keywords:** acceptance and commitment therapy, chronic kidney disease, health psychology, hemodialysis, depression

## Abstract

Depression is a prevalent issue among patients with chronic kidney disease, particularly those undergoing hemodialysis. Its presence is associated with reduced quality of life, poor treatment adherence, and an increased risk of hospitalization and clinical deterioration. There is a pressing need for further research into effective psychological interventions aimed at improving the mental health of this population. This open-label pilot clinical trial involved five hemodialysis patients residing in Madrid who had been receiving treatment for over 3 months and exhibited clinically significant depressive symptoms. The intervention consisted of eight weekly, individual, 60-min face-to-face sessions based on a structured Acceptance and Commitment Therapy (ACT) protocol, delivered during dialysis. Outcomes were assessed using standardized questionnaires measuring quality of life, psychological symptoms, psychological inflexibility, and coping strategies at four time points: pre-treatment, post-treatment, and at 3- and 8-month follow-ups. A within-subject analysis was conducted, and the Reliable Change Index was applied to evaluate clinical significance. All participants completed the intervention, which was well accepted and deemed feasible, with high levels of satisfaction reported. Sustained reductions in depressive symptoms were observed in all participants, and improvements in quality of life were noted in three. No significant changes were found in psychological inflexibility or coping strategies. This pioneering pilot study in a Western context provides preliminary evidence supporting the feasibility and potential effectiveness of ACT for treating depression in hemodialysis patients. It highlights the viability of delivering psychological interventions within dialysis units and suggests ACT as a promising therapeutic approach for this population.

## 1 Introduction

Chronic kidney disease (CKD) is considered a silent epidemic due to its high impact on Public Health, affecting 15.1% of the population (Gorostidi et al., 2018). In the last decade, the number of new patients requiring renal replacement therapy in Spain has increased by 11.6% to reach 141.4 people per million population in 2020 (Sociedad Española de Nefrología, 2022). It has a higher incidence and comorbidity with diabetes mellitus, hypertension, older people, women and racial minorities, being one of the causes of the highest mortality in the world and as well as loss of quality of life (QoL), early retirement and work incapacity (Martín and Lorenzo, 2024). CKD, hemodialysis (HD) and the uncertainty associated with the transplant waiting list lead to significant losses and serious stress in patients, which affect the quality of life and have emotional and behavioral consequences (Shery et al., 2019; Marín et al., 2022), demanding the ability to adjust to changes (Aránega-Gavilán et al., 2022; Heilman et al., 2017).

Depending on the assessment method used, depression is estimated to be present in 21–39% of renal patients (Marín et al., 2022; Palmer et al., 2013). Its presence is associated with the use of steroids and immunosuppressive drugs, female gender, older age, lower educational level, and low QoL, and is more likely if HD is used and also the longer the period of time on dialysis (Ćwiek et al., 2017; Drayer et al., 2006; García-Llana et al., 2014; Vázquez-Martínez et al., 2016). Depression is associated with poorer adherence to treatment and a higher risk of hospitalization and deterioration (Erbay et al., 2021) and is the greatest predictor of health-related QoL (Marín et al., 2022). Depression predicts mortality in CKD after adjusting for age, sex, race, and medical comorbidities (Drayer et al., 2006).

Cognitive-behavioral psychological therapies (CBT) have demonstrated evidence in their application to CKD, improving self-efficacy and self-care (Kim, 2018; Yang et al., 2015; Weiner et al., 2010), reducing depressive symptoms associated with the course of the disease and HD, and improving adherence to pharmacological treatment and the establishment of dietary preventive measures such as fluid restriction or reduction of salt intake (Othman et al., 2020; Nozaki et al., 2005; Payne et al., 2013). Research has revealed certain limitations in the effectiveness of CBT interventions, particularly with regard to reducing the perception of pain, disability, and emotional distress in chronic diseases (Williams et al., 2020).

Ground-breaking therapeutic approaches are being explored in the field of health psychology that seek to promote acceptance and psychological flexibility (PF) to transform the relationship with emotional discomfort, thus facilitating more effective, functional, and sustainable behavioral change processes over time. PF has been defined as being in touch with the present moment without defense and persisting in a behavior or changing it based on chosen values (Hayes et al., 2012). Among the most innovative approaches with the greatest empirical support is Acceptance and Commitment Therapy (ACT), a third-generation cognitive-behavioral therapy based on functional contextualism and relational frame theory (Hayes, 2016). Given that renal patients face a context of chronic loss and uncertainty, unlike CBT approaches—which are more focused on cognitive and emotional control through promoting positive emotions and thoughts—ACT, which assumes that the human ability to arbitrarily relate verbal events is intrinsic to our condition, emphasizes psychological acceptance and flexibility. Therefore, it may offer these patients effective skills to openly and mindfully engage with feelings of loss and future-related concerns inherent to the illness. At the same time, it may help them remain focused and active in vital life domains such as relationships with partners, family, and friends, health, and treatment adherence, maintaining balance among these important areas. In this way, the experience of suffering associated with the loss of health can be reframed as an opportunity for personal growth (Hayes, 2019; Kashdan and Rottenberg, 2010).

Acceptance and Commitment Therapy has been shown to be effective in addressing different psychological problems such as anxiety, stress, depression, obsessive-compulsive disorder and drug abuse (Gloster et al., 2020). In health psychology, its effectiveness has been proven in different chronic diseases to improve the QoL and reduce the symptoms of anxiety and depression (Konstantinou et al., 2023), highlighting its development in chronic pain (Feliu-Soler et al., 2018) and psycho-oncology (Zhao et al., 2021). In CKD, ACT has revealed its contribution to improving emotional well-being (Arnout, 2019), quality of life (Kalbasi et al., 2021; Rouhi et al., 2023), disease perception (Karimi et al., 2019), therapeutic adherence (Rzeszut, 2011; Sadeghi et al., 2024), resilience (Tajbakhsh et al., 2023), emotional well-being (Sarizadeh et al., 2019), spiritual health (Kazemi Daluee and Samari, 2021), decreased depression (Azemi Zeynal et al., 2016), fatigue and enhanced PF (Elander et al., 2023), most studies having been conducted with non-Western patient populations. Although ACT is a promising approach that has already been recognized as a treatment with strong empirical support in applications in health psychology such as chronic pain (Feliu-Soler et al., 2018), there is a lack of studies with CKD in a Western population that evaluate the feasibility and usefulness of ACT on depressive symptoms. Studies are needed that adapt ACT to the needs of Spanish patients on HD with depression. This pilot study aims to explore the feasibility and potential utility of ACT in chronic kidney patients undergoing dialysis residing in Spain with significant levels of depression. It also seeks to evaluate the relevance and practicality of conducting a clinical trial on this topic. Reductions in depression and emotional distress and improvement in QoL were expected after treatment. Changes in symptomatology were expected to be accompanied by changes in processes such as psychological inflexibility and coping strategies. Another objective is to assess whether psychological intervention could be delivered during treatment at the hemodialysis ward, which could lead to greater acceptability. Furthermore, this study is part of a larger project aimed at obtaining evidence to justify conducting a randomized clinical trial with a larger sample. Given the lack of controlled studies in Western populations, we developed this open-label pilot trial as an initial step to evaluate the feasibility and acceptability of an ACT intervention. These results will inform the design of a fully powered randomized controlled trial that directly addresses this gap.

## 2 Method

### 2.1 Design

Open-label pilot clinical trial with no control group. Measures were collected using standardized questionnaires before the intervention, at post-treatment and at 3-month and 8-month follow-up, to evaluate both medium-term and longer-term maintenance of treatment effects. The 3-month time point allowed us to assess the stability of immediate gains, while the 8-month follow-up provided insight into whether improvements persisted over time in the context of a chronic health condition. These time frames were also feasible within the organizational context of the dialysis units and maximized retention given the clinical vulnerability of the sample.

### 2.2 Participants

**Figure 1:**
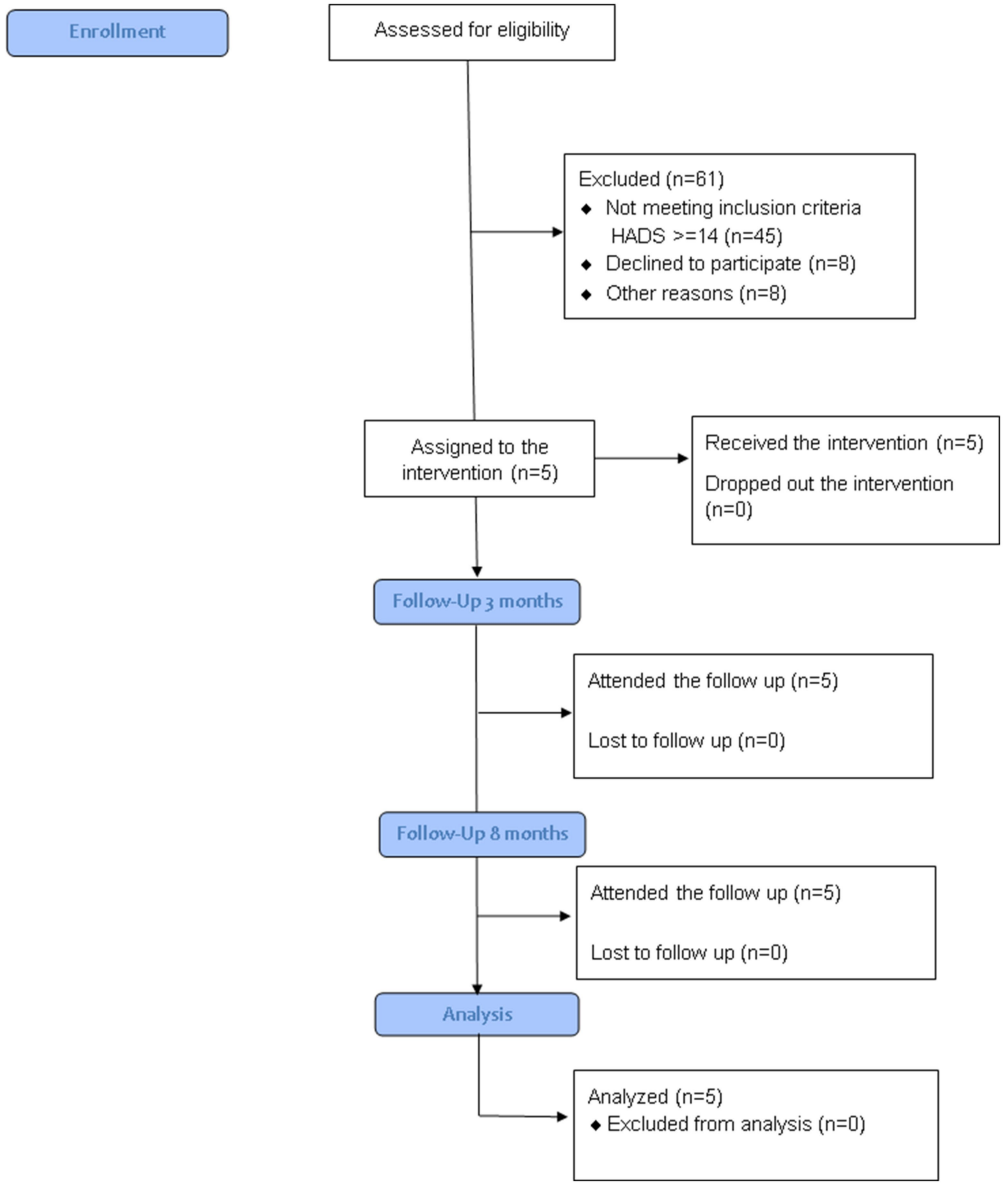
Consort flow diagram.

The study included five patients diagnosed with CKD who were undergoing HD at the Santa Engracia Dialysis Center in Madrid, operated by the Fundación Renal Española. The sample comprised four men and one woman, with a mean age of 51.6 years (SD = 7.26). Only one participant was actively employed, while the remaining four were retired. The average duration on dialysis was 2.6 years. Inclusion criteria were (1) on HD for at least 3 months, (2) total score on HADS ≥14, following the criteria suggested by Preljevic et al. (2012) and (3) clinically significant levels of depression. Exclusion criteria were (1) diagnosis of severe mental disorder, (2) cognitive impairment, (3) on psychiatric treatment or with antidepressant drugs and (4) advanced disease. As shown in Figure 1, a total of 66 patients were evaluated as candidates for the study, of which only 5 met the inclusion criteria and constituted the final sample for this study.

### 2.3 Outcomes

The primary outcomes were QoL, depression and emotional distress assessed through standardized questionnaires. Secondary outcomes evaluated anxiety, psychological inflexibility and coping strategies through standardized questionnaires. Additional secondary outcomes included treatment satisfaction, which was assessed using a questionnaire specifically developed for the present study. This *ad hoc* instrument comprised Likert-scale items evaluating overall usefulness, perceived support, communication with the therapist, the weekly exercises, and perceived difficulty.

### 2.4 Measures

Anxiety, depression and emotional distress measured by the *Hospital Anxiety and Depression Scale (HADS)*. It is a 14-item four-point Likert-type scale. Scores range between 0 and 42. Higher scores in the overall score indicate higher levels of emotional distress. It includes two subscales, which, respectively, assess generalized anxiety and depression. By avoiding somatic symptoms like fatigue or insomnia, which can coexist with physical disorders, the HADS ensures a more accurate evaluation of psychological symptoms. Designed for chronic patients, it has been validated in Spanish patients (Herrero et al., 2003) demonstrating adequate reliability and validity.

Health Related Quality of Life (QoL) measured by the *COOP/WONCA questionnaire*, composed of 9 sheets commonly used in primary care and adapted to Spanish CKD patients with adequate reliability and validity (Arenas et al., 2004). Cartoon drawings are included in the questionnaire to make it simple for patients to comprehend and rate their current state of health. The 7-item five-point Likert-type version of the scale was selected. Scores range between 0 and 35. Higher scores indicate lower levels of QoL.

Psychological inflexibility (PI) measured by the Spanish version of the *Acceptance and Action Questionnaire-II* adapted to the context of patients undergoing hemodialysis (AAQHD-II) (Domínguez et al., 2020). PI is a persistent pattern of behavior characterized by avoidance of discomfort that interferes with the achievement of valued goals. It is a 7-item, seven-point Likert-type scale. Scores range between 7 and 49. Higher scores indicate higher levels of PI. Published studies have shown adequate validity and reliability in Spanish hemodialysis patients (Domínguez et al., 2020).

Coping strategies measured through the *Coping Strategies Inventory Short-Form* (CSI-SF). It is a 15-item, five-point Likert-type scale that includes 4 subscales: Problem Focused Engagement (PFE), Problem Focused Disengagement (PFD), Emotion Focused Engagement (EFE) and Emotion Focused Disengagement (EFD). Engagement scales refer to exposure to stressors, while disengagement scales allude to avoidance. The Spanish adaptation showed adequate reliability and validity (Tous-Pallarés et al., 2022).

Satisfaction with therapy, evaluated using a custom-designed *Therapy Satisfaction Questionnaire*, assessing the perceived usefulness of treatment strategies, difficulty and the overall satisfaction with therapy on a scale of 1 to 10.

### 2.5 Procedure

The study was authorized by the Ethics Committee of the Fundación Jiménez Díaz. All participants signed an informed consent form and the voluntary nature of their participation and confidentiality of the data were guaranteed. Recruitment, assessment, and intervention were carried out while the patients were undergoing hemodialysis at the Fundación Renal Española centers in Madrid. At the beginning, an evaluation session was carried out through a semi-structured interview, which allowed for the functional analysis and adaptation of the intervention to each patient. The intervention included a closed protocol applied over 8 individual face-to-face 60-min sessions on a weekly basis based on ACT (Hayes et al., 2012). Although administered flexibly, the intervention in all patients included the same therapeutic methods applied in the same order. A summary of the clinical methods in each session can be found in Table 1. The intervention protocol is available to interested parties upon reasonable request. The intervention was administered by one therapist with specialized training in ACT, the first author supervised by the study director (last author). Measures were collected at pre and post-treatment and at 3 and 8-month follow-up. Given that patients undergoing hemodialysis frequently experience fatigue, if a patient reported feeling unwell or lacking energy at the scheduled time for an assessment or intervention session, the session was postponed to the next available day for the researcher. This decision was made only after ensuring that the rescheduling would not inadvertently reinforce the patient’s avoidance behaviors.

**Table 1:** Summary of the intervention protocol.

Therapeutic methods	Therapeutic processes						Sessions							
	Values	Acceptance	Defusion	Commitmed action	Attention to present moment	Self as Context	1	2	3	4	5	6	7	8
“Gardening” metaphor (Hayes et al., 2012)	X	X		X			X	X						X
Defining committed actions				X			X	X	X	X	X	X	X	X
“Man in the Hole” Metaphor (Hayes et al., 2012)		X						X						
“Do not think of”… exercise [adapted from Chocolate cake exercise (Hayes et al., 2012)]		X							X					
“Polygraph” metaphor (Hayes et al., 2012)		X							X					
“Fall in love at will” exercise		X							X					
“Rule of 95–5%” (Wilson and Luciano, 2002)		X												
“The Ambulance” metaphor, adapted from “Passengers on the Bus” metaphor (Hayes et al., 2012)			X			X				X	X			
“Bodyscan” exercise (Kabat-Zinn, 1990)			X		X	X					X			
“Physicalizing” exercise (Hayes et al., 2012)			X			X					X			
“Noticing sensations in the room” exercise			X	X		X					X			
“Contents on cards” exercise (Ruiz et al., 2018)			X			X						X		
“Thoughts in clouds” exercise [adapted from “Soldiers in the parade” exercise (Hayes et al., 2012)]			X		X	X						X		
Work on the highest-ranking private events (Big Ones) [adapted from Ruiz et al. (2018)]			X										X	
Exposure to thoughts: therapist acting as client’s mind while client stays focused in a simple task [adapted from “Taking your mind for a walk” (Hayes et al., 2012)]			X			X							X	
“Falling of the bike” metaphor [adapted from “The rider” metaphor (Hayes et al., 2012)]	X	X												X
“Fall and leaves” exercise (Wilson and Luciano, 2002)			X		X	X								X

### 2.6 Statistical analysis

Considering the small number of participants, a within-subject analysis was performed. To determine whether the psychological intervention was clinically significant, the Reliable Change Index (RCI) was used for the dependent variables. The researchers used the method proposed by Jacobson and Truax (1991), which allows observing whether the change in the variables collected is clinically significant and reliable. Three different criteria are proposed by the authors, each with its respective cut-off points. In this study, Criterion A was selected — defined as a post-treatment score falling two standard deviations beyond the mean of the clinical sample — because it best fits the data structure and design of this pilot trial. They maintain that this occurs when there is a return to normal functioning and the change becomes part of the functional population (Castillo, 2010). Three different criteria are proposed, each with its respective cut-off points. The first criterion is the one that best fits this study: a point of two standard deviations beyond the range of the mean in the pre-measure [Cutoff A (Jacobson and Truax, 1991)]. For the analysis of the effect of the intervention, the global scales of the variables were considered.

## 3 Results

### 3.1 Adherence and feasibility

The intervention had a high adherence rate. None of the participants dropped out of the intervention. All five patients completed the eight intervention sessions and provided all requested data both at post-treatment and at 3 and 8 month follow-up. Satisfaction with the intervention was high (mean: 8.6 out of 10). The most highly valued aspect was the support obtained from the therapist (9.4), followed by communication with the therapist (9), weekly tasks (8.4) and therapeutic methods (8). The average perceived difficulty was 6.4 and the overall usefulness 8.4. The implementation of the intervention showed its feasibility and acceptability. The study also showed the feasibility of administering the individual intervention to patients in the dialysis room. Patients readily accepted the loss of privacy that came with receiving the intervention in this space and thus not having to spend more time in the healthcare center. The initially planned duration of the 60-min sessions proved to be slightly excessive, as most patients were able to maintain attention for only 50 to 55 min. Consequently, the session content was adjusted and condensed accordingly to better align with participants’ attention spans.

### 3.2 Within-subject analysis

**Figure 2:**
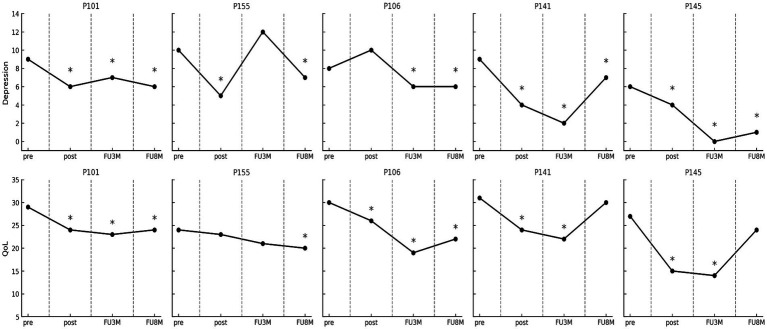
Evolution in depression and quality of life measures in every participant. Pre, pre-treatment; Post, post-treatment; FU3m, three-month follow-up; FU8m, 8-month follow-up. COOP-Wonca score reductions reflect QoL improvements. * indicates a clinically significant recovery according to criterion “A” of the Jacobson and Truax method (Jacobson and Truax, 1991).

Figure 2 shows the temporal evolution of the direct scores on the depression and QoL measures in each of the participants. The results regarding the clinical significance of the changes after the intervention evaluated through standardized questionnaires revealed by the RCI are shown in Table 2.

**Table 2:** Clinical significance of changes in measures through the reliable change index (RCI).

Variables	Timepoint comparisons	Participants				
		P101	P155	P106	P141	P145
QoL	Pre-post	−2.605*	−0,521	−2.084*	−3.648*	−6.253*
	Pre-FU 3 m	−3.126*	−1.563	−5.732*	−4.690*	−6.774*
	Pre-FU 8 m	−2.605*	−2.084*	−4.169*	−0.521	−1.563
Emotional distress	Pre-post	−8.389*	−3.728*	1.864	−7.451*	−9.321*
	Pre-FU 3 m	−7.457*	5.592^#^	−4.660*	−10.253*	−13.981*
	Pre-FU 8 m	−9.321*	−1.864	−4.660*	−2.796*	−12.117*
Anxiety	Pre-post	−3.39*	0.565	0	−1.695	−4.52*
	Pre-FU 3 m	−3.389*	2.259^#^	−1.694*	−2.259*	−5.084*
	Pre-FU 8 m	−3.955*	0.565	−1.695	−0.565	−4.52*
Depression	Pre-post	−3.223*	−5.372*	2.149*	−5.372*	−2.149*
	Pre-FU 3 m	−2.149*	2.149^#^	−2.149*	−7.520*	−6.446*
	Pre-FU 8 m	−3.223*	−3.223*	−2.149*	−2.149*	−5.372*
PI	Pre-post	−0.238	0.238	0.476	−1.906	−3.514*
	Pre-FU 3 m	−0.468	4.764^#^	−2.144*	−1.667	−2.858*
	Pre-FU 8 m	−0.953	3.335^#^	−0.715	−0.715	1.191
PFE	Pre-post	0.794	3.175*	0.397	1.588	−2.381^#^
	Pre-FU 3 m	0	1.985*	0.397	0.794	0.397
	Pre-FU 8 m	1.191	2.381*	0.397	0.397	−1.191
EFE	Pre-post	0	4.227*	−0.384	0	−3.044^#^
	Pre-FU 3 m	−0.384	1.921	0	0.384	−1.153
	Pre-FU 8 m	−1.153	3.843*	0	−0.769	−3.074^#^
PFD	Pre-post	0	2.691*	1.076	0	−1.614
	Pre-FU 3 m	−0.538	6.458*	−0.538	0	−0.538
	Pre-FU 8 m	0.538	6.996*	0	−0.538	1.614
EFD	Pre-post	−0.955	1.911	0.478	0	−3.821*
	Pre-FU 3 m	−2.866*	2.866^#^	0	−1.433	−4.299*
	Pre-FU 8 m	−1.433	3.344^#^	1.433	−0.478	−1.911

Pre, pre-treatment; Post, post-treatment; FU 3 m, three-month follow-up; FU 8 m, 8-month follow-up; QoL, quality of life; PI, psychological inflexibility; PFE, problem focused engagement; EFE, emotion focused engagement; PFD, problem focused disengagement; EFD, emotion focused disengagement. *Indicates a clinically significant and reliable change (Cutoff A). ^#^Indicates a clinically significant and reliable worsening (Cutoff A).

The QoL showed clinically significant improvement in four of the five patients both at the end of the intervention and at the three-month follow-up. Furthermore, three participants maintained this improvement at 8 months. The intervention was followed by a clinically significant decrease in depression levels in all patients at the end of the intervention. While these effects were maintained in four of them at the three-month follow-up, one of them (P155) worsened. However, all patients, including the one who worsened at 3 months, showed clinical recovery at 8 months.

Psychological treatment was followed by a reduction in overall anxiety levels. Two participants achieved clinically significant improvements at the end of the intervention, and this number increased to four at the three-month follow-up. However, one participant showed clinical worsening during this period. These improvements in anxiety were maintained at 8 months in two participants. Regarding emotional distress, which was considered one of the inclusion criteria, four participants showed clinically significant improvements at post-treatment, at three and 8 months. In this case, one of the participants who had improved (P155) worsened at the three-month follow-up and showed no change from pre-intervention levels at 8 months. However, the participant who did not show improvements at post-treatment (P106) did show improvements at both follow-ups.

Regarding IP, one participant improved after the intervention, two improved at 3 months, but one returned to pre-treatment levels. At 8 months, the changes were not maintained, and one participant worsened. For adaptive coping strategies related to engagement, one participant improved in PFE and one worsened after the intervention. At the three-and eight-month follow-ups, only one participant maintained recovery. In terms of EFE, one participant improved and one worsened after the intervention. There were no changes at 3 months, but at 8 months, one participant improved and one worsened. For maladaptive disengagement coping strategies, one participant improved in PFD after the intervention, maintaining the effects at 3 and 8 months. One participant showed recovery in EFD after the intervention, two at 3 months, but one worsened. At 8 months, one participant worsened.

### 3.3 Main changes reported by patients

All patients reported greater satisfaction with their health management, and four of them improved their satisfaction with family, friendship, and leisure activities. Regarding behavioral changes, all patients reported spending more time with family and friends after the intervention, and four of them increased their frequency of physical activity and improved their eating and fluid intake habits.

## 4 Discussion

This pilot study provides preliminary evidence for the feasibility and usefulness of a brief Acceptance and Commitment Therapy (ACT)-based protocol in patients with Chronic Kidney Disease (CKD) undergoing hemodialysis who present clinically significant depressive symptoms. Despite the inherent limitations of pilot studies, the findings offer a positive perspective on the intervention and pave the way for larger-scale research. In terms of acceptability and adherence, the results were exceptional. All patients completed the treatment protocol without dropping out of the study, and high levels of satisfaction with the intervention were reported. This is particularly relevant, as it demonstrates that dialysis patients—often perceived as having low willingness to engage in psychological interventions—can benefit from such therapies even in a clinically complex environment like the hemodialysis room. This high adherence may be related to the fact that the intervention was delivered within a setting already integrated into the patients’ routine, thus removing logistical barriers such as transportation or the stress associated with attending additional healthcare appointments to receive psychological treatment.

Regarding the clinical outcomes, a significant improvement in QoL and a reduction in depressive and anxiety symptoms were observed in the majority of patients, with these improvements maintained at the eight-month follow-up in a considerable proportion of the sample. This suggests that the ACT-based protocol may have sustainable effects, even in the absence of continuous therapeutic support. The decrease in emotional distress in four out of the five patients is another positive indicator, suggesting that the intervention may have been associated with improved emotional well-being, although causal conclusions cannot be drawn in the absence of a control group.

The fact that some patients, such as P155, experienced a temporary worsening of depression and anxiety at the three-month follow-up, followed by an improvement at 8 months, reflects a possible intermediate relapse that may be common in patients with chronic illnesses. The patient’s lack of short-term response to treatment may have been hypothetically attributed to challenges within his family context. During the week of the second follow-up, he disclosed having received distressing news regarding his mother’s health. He reported experiencing considerable emotional turmoil, including sadness and frustration, stemming from his inability to travel to his home country to visit her. In any case, this also suggests that, while the intervention may have contributed to recovery, the process of emotional adaptation to a chronic illness like CKD can be non-linear, with ups and downs over time and highly influenced by the patients’ personal context, due to their special vulnerability. This pattern could be interpreted as a reminder that emotional management in patients with chronic illnesses often requires ongoing interventions and adjustments over time.

Improving QoL, rather than symptom reduction, is considered the overall aim of ACT and its improvement after ACT administration is in line with the evidence found in previous studies in a wide range of chronic health conditions (Konstantinou et al., 2023) and CKD patients (Kalbasi et al., 2021; Rouhi et al., 2023), and may be related to the fact that the intervention is focused on improving functioning and not so much on alleviating discomfort. Likewise, the results are in line with previous studies that have shown a reduction in depression in chronically ill patients (Konstantinou et al., 2023) and CKD patients (Azemi Zeynal et al., 2016). A desirable and noteworthy outcome is the sustained improvement over time (Hoffmann et al., 2018), which could be a consequence of the continued practice of the acquired skills. This is an encouraging result that suggests the potential usefulness of the intervention; however, caution is warranted due to the absence of a control group and the small sample size.

Changes in both QoL and depression may be related to the fact that therapy motivates patients to take action and introduce changes in their daily lives (Konstantinou et al., 2023). Since the goal of ACT is not to reduce distress or depressive mood, but rather to ensure that the patient remains active regardless of mood fluctuations (Hayes et al., 2012), the reduction of depressive symptoms is not a primary goal of therapy, although such reduction may be an effect of newly acquired skills. Thus, the goal of accepting CKD and living with the disease becomes equivalent to acting to actively and continuously cultivate the different valued facets and, in particular, to addressing the risk of abandoning facets related to leisure, interpersonal relationships, and health or self-care, whether or not the discouragement or worry associated with CKD is present. In this sense, it is possible that, although no direct changes in PI or coping strategies were observed, the intervention may have contributed to patients learning to continue behaving actively, which could have led to the observed improvements in QoL and reduction in depressive symptoms. The focus for adapting to the chronic illness condition would be on improving functioning and maintaining balance among important life domains. This goal could have been achieved by systematically defining committed actions and inviting patients to implement these commitments between sessions, specifying details such as the day, place, or people with whom they would carry out the actions.

A notable and innovative aspect of this study is the fact that patients received psychological treatment during hemodialysis. This context for administering the psychological intervention represents a considerable advantage that may have contributed to the excellent adherence, as travel did not represent additional stress for patients already burdened by significant treatment-related burdens. Similarly, administering an ACT-based intervention in a setting where many triggers of distress are present (e.g., awareness of illness while undergoing hemodialysis, feelings of lack of freedom associated with the time spent on hemodialysis, low blood pressure, witnessing health crises in other patients, learning about colleagues whose health is deteriorating or who have died, etc.) may have provided valuable opportunities for emotional exposure and the practice of emotion regulation skills. However, further research is needed to evaluate the usefulness of ACT in managing the impact of these contextual factors. Even so, it was a significant challenge for the therapist to operate in the presence of a multitude of noises from the dialysis machines and interference related to medical procedures and the work of other professionals sharing the space, although the therapeutic benefits outweighed the discomfort this situation caused for the therapist. This characteristic, along with the abbreviated format of the intervention, represented considerable advantages of the intervention studied.

### 4.1 Limitations

The small number of participants, mostly middle-aged men, prevents the results from being generalized to the CKD patient population. The measures used were exclusively self-report, which may lead to biases related to social desirability, subjectivity, or difficulties in detecting or remembering changes. It would be desirable in future studies to complement the self-report measures with objective measures related to behavioral changes, for example, collected through self-monitoring or external observers, as well as other medical indicators.

Given that a number of patients declined to participate in the study, it can be assumed that those who agreed were those who showed greater motivation to receive psychological support, and the sample selection may have shown this bias toward patients with a particular motivation for treatment. This sample selection bias could be reduced by conducting studies with larger samples that included patients with different levels of motivation.

Future research exploring effects of ACT on CKD should include more controlled studies with larger samples. In any case, given the complexity and vulnerability of this patient population, small-N feasibility studies represent a necessary preliminary step before progressing to controlled trials. This approach aligns with prior and ongoing ACT-based pilot studies conducted in clinical populations facing comparable challenges (Hoffmann et al., 2018; Meuret et al., 2012; Martin et al., 2016). Although the limited sample size in the present study precludes generalizability, the observed improvements suggest that brief ACT interventions may represent a promising strategy for reducing depression in this population. These findings, while preliminary, should be interpreted with caution. Nevertheless, they provide a valuable foundation for the design of a fully powered randomized controlled trial to evaluate the intervention’s efficacy under more stringent conditions. This pilot study supports the rationale for our team to initiate such a trial. Randomized controlled trials might compare the effectiveness of ACT versus CBT, face-to-face versus online delivery of ACT, and individual versus group-based ACT formats. Future studies could also explore the impact of psychological interventions on medical therapeutic adherence and CKD symptoms and CKD symptoms. Health psychologists could use this information to plan, improve and implement additional interventions. Specifically, we strongly recommend exploring the usefulness of an intensive ACT-based approach in predialysis preventive interventions to promote patient adherence and well-being before initiating a treatment as demanding as renal replacement therapy. We must not forget that by striving to improve adherence and well-being in kidney patients, we facilitate a more rational use of resources.

In summary, despite the small number of participants, this contribution strengthens person-centered care (Morton and Sellars, 2019), lays the groundwork for expanding the range of available psychotherapeutic approaches, and enhances the interdisciplinary management of CKD. It represents a new way of understanding the design and delivery of services for people with kidney disease that focuses on functioning rather than emotional symptoms and can be administered in the dialysis room, representing a shift from the way nephrology services have historically been designed. This study highlights the importance of implementing a person-centered approach to renal patients undergoing hemodialysis. It is essential to recognize that the technical skills required to resolve issues such as a central venous catheter are equally important as the competencies needed to address the emotional distress of patients. We should not forget that skills for unblocking a problem with a central venous catheter are just as important as those for unblocking the emotional state of a distressed patient. Ultimately, the implementation of an ACT-based psychological intervention during hemodialysis for patients experiencing depression related to kidney disease appears to be a potentially valuable therapeutic approach. However, determining its effectiveness warrants further investigation under more rigorous experimental conditions.

## Data Availability

The raw data supporting the conclusions of this article will be made available by the authors, without undue reservation.
